# Longitudinal Effects on Metabolic Biomarkers in Veterans 12 Months Following Discharge from Pharmacist-Provided Diabetes Care: A Retrospective Cohort Study

**DOI:** 10.3390/pharmacy10030063

**Published:** 2022-06-13

**Authors:** Candis M. Morello, Lytia Lai, Claire Chen, Chui Man Leung, Jan D. Hirsch, Mark Bounthavong

**Affiliations:** 1 Division of Clinical Pharmacy, Skaggs School of Pharmacy and Pharmaceutical Sciences, University of California San Diego, La Jolla, CA 92093, USA; lytia.lai@gmail.com (L.L.); claire.chen2426@gmail.com (C.C.); mandyleung4391@gmail.com (C.M.L.); mbounthavong@health.ucsd.edu (M.B.); 2 Department of Pharmacy, Veterans Affairs San Diego Health System, San Diego, CA 92161, USA; 3 Department of Pharmacy, Veterans Affairs Greater Los Angeles, Los Angeles, CA 90073, USA; 4 Department of Pharmacy, Sharp Chula Vista Medical Center, Chula Vista, CA 91911, USA; 5 Department of Clinical Pharmacy Practice, School of Pharmacy and Pharmaceutical Sciences, University of California Irvine, Irvine, CA 92612, USA; jdhirsch@uci.edu

**Keywords:** long-term effects, collaborative practice, clinical pharmacy, diabetes, pharmacist, comprehensive medication management (CMM), patient care management, ambulatory care, veterans

## Abstract

Clinical pharmacist interventions have resulted in optimized diabetes control in complex patients; however, there are no studies examining the durability of achieved outcomes after patients discontinued being seen by the pharmacist. A pharmacist-led comprehensive medication management (CMM) Diabetes Intensive Medication Management (DIMM) “tune up” clinic provided the opportunity to evaluate long-term glycemic control outcomes following clinical discharge. This study used a retrospective cohort study design with a matched primary care provider (PCP) comparison group. Outcomes were compared between the groups at several post-discharge intervals (6, 9, and 12 months) using independent t tests and chi-square tests, where appropriate. DIMM-managed patients achieved an average HbA1c reduction of 3% upon discharge, and maintained an average HbA1c concentration that was significantly lower than PCP-managed patients at 6 months (*p* < 0.001) and 9 months (*p* = 0.009) post-discharge. Although DIMM-managed patients had lower HbA1c than PCP-managed patients at 12 months post-discharge, the difference was not significant (*p* = 0.105). Similar findings were noted for average FPG and LDL across the study time points. No differences in average HDL levels were reported across the time points. A significantly larger proportion of DIMM-managed patients maintained HbA1c < 8% compared to PCP-managed patients at 6 months (67.5% versus 47.2%, *p* = 0.001) and 9 months (62.6% versus 40.6%, *p* = 0.040) post-discharge; DIMM-managed patients had a larger, but non-significant, proportion of goal retention compared to PCP-managed patients at 12 months (56.9% versus 47.2%, *p* = 0.126) post-discharge. Similarly, a significantly larger proportion of DIMM-managed patients sustained HbA1c < 9% compared to PCP-managed patients at 6 months (87.8% versus 66.7%, *p* < 0.001) and 9 months (82.1% versus 68.3%, *p* = 0.012) post-discharge; however, there was no significant difference at 12 months. The attenuation of the DIMM-managed metabolic biomarkers suggests that an additional follow-up visit or touchpoint may be helpful. The personalized care of the DIMM “tune up” approach was successful in achieving sustained glycemic control for up to 9 months. Outcomes can help inform future long-term result durability evaluations.

## 1. Introduction

In the United States (US), diabetes is the 7th leading cause of death (1999–2020) and affects approximately 34.1 million people, or 13.0% (10.2% diagnosed and 2.8% undiagnosed) of the total adult (18 years or greater) population [1,2]. Diabetes is associated with comorbidities and complications such as chronic kidney disease, vision disability, as well as hospitalizations for major cardiovascular disease, lower extremity amputation, hyperglycemic crisis, and hypoglycemia [2]. Careful control of metabolic and cardiovascular end points can significantly reduce diabetes-associated morbidity and mortality. However, patients with diabetes can be challenging to manage. High medication burden and other comorbidities require intensive medication management to reduce the potential for non-adherence, drug–drug interactions, and costly microvascular and macrovascular complications including, but not limited to, heart attack, stroke, kidney problems, vision loss, nerve damage, and poor oral health [3,4,5,6,7,8]. Among veterans, the prevalence of diabetes is much higher than in the general population (20.5% diagnosed and 3.4% undiagnosed), and the case mix is often more complex, which has generated demand for an intensive diabetes management clinic at the US Veterans Health Administration (VA) [9]. In response to this need, and in collaboration with the endocrinology department, the pharmacy department at the VA San Diego Healthcare System (VASDHS) implemented a pharmacist-led Diabetes Intense Medication Management (DIMM) “tune up” clinic to manage complex cases of veterans with type 2 diabetes [10,11,12]. 

The DIMM “tune up” clinic model provides comprehensive medication management (CMM), which is a systematic approach to medications where physicians and pharmacists work together to ensure that all medications (e.g., prescription, nonprescription, alternative, traditional, vitamins, or nutritional supplements) are individually assessed to determine that each medication is appropriate, effective for the medical condition, safe for the patient (given comorbidities and concurrent use of other medications), and can be taken by the patient as intended [13]. In addition, the focus is on achieving personalized clinical goals of therapy by determining drug therapy problems, developing an individualized care plan, and assessing clinical outcomes over time.

The DIMM “tune up” clinic is operated one-half day per week and is led by clinical pharmacy specialists (CPS) who are authorized to make clinical interventions to optimize diabetes control and associated co-morbid conditions in complex patients under a collaborative agreement with an endocrinologist and offers a limited number of 60-min visits that combine CMM with patient-specific diabetes education. The scope of practice for a CPS includes independently assessing lab results and prescribing and adjusting diabetes and co-morbid conditions and medications, in addition to providing personalized education. DIMM clinic patients are regularly scheduled for follow-up visits in 2–3 month increments with the aim of reaching their personalized hemoglobin A1c (HbA1c) goal within 6 months. To motivate patients and help them take control of their diabetes with long-term healthy lifestyle and behavior changes, between visits, patients were given personalized tools, including short phone visits initiated by the patient to evaluate therapy response and dose titrations. Once patients’ glycemic goals were achieved, they were discharged from the DIMM “tune up” clinic and returned to the care of their primary care provider (PCP). A full description of clinic development, CPS responsibilities, patient specific care provided, and initial 6-month outcomes was previously published [11]. 

Comparative effectiveness studies comparing the pharmacist-led DIMM “tune up” clinic to the standard of care by primary care providers reported significant improvement in the HbA1c end point among veterans. In a 2016 study, Morello and colleagues reported that the percentage of patients who achieved their personalized HbA1c goal (<7%, <8%, <9%) at 6 months was significantly greater in the DIMM group compared to typical care in a PCP comparison group for all comparisons [11]. In addition to clinical efficacy, the DIMM clinic was estimated to have lower cost for a greater gain in quality-adjusted life year compared to the PCP comparison group over the 2-, 5-, and 10-year time horizons, with a return on investment of $9.01 per dollar spent [14]. Although these studies demonstrated a positive impact while attending the DIMM clinic, none examined the durability of achieved outcomes after patients discontinued being seen by the pharmacist. Other studies have reported improvement in HbA1c reduction among patients with diabetes who were managed in a pharmacist-led diabetes management clinic [15,16,17]. However, these studies lacked a control group, which would have ruled out alternative explanations for the observed reduction in HbA1c concentrations, and none examined the durability of the achieved response after patients discontinued clinical treatment. To address these gaps, we sought to evaluate the long-term impact of the DIMM “tune up” clinic among patients who had achieved (at discharge) their HbA1c goals 12 months after they were discharged back to their PCPs. 

The primary study objective was to compare the long-term glycemic efficacy outcomes (HbA1c) spanning 12 months post-discharge from the DIMM “tune up” clinic against a matched control group of patients who had their diabetes managed by their primary care provider (PCP-managed group) during the same period. Secondary objectives compared the long-term impact of the DIMM “tune up” clinic on proportions of patients achieving glycemic goals (<7%, <8%, <9%) and on other metabolic parameters, such as fasting plasma glucose (FPG), low-density lipoproteins (LDL), and high-density lipoproteins (HDL), between groups. 

## 2. Materials and Methods

### 2.1. Study Design

A retrospective cohort design was used to evaluate the long-term (12 months) glycemic and metabolic outcomes (HbA1c, LDL, HDL, and FPG) of complex patients with diabetes at the VASDHS who received care in the DIMM “tune up” clinic between April 2009 and October 2019 and were discharged back to their primary care providers, compared to typical care within the PCP-managed group. We followed the Strengthening the Reporting of Observational Studies in Epidemiology (STROBE) statement for describing our study design and reporting our findings [18]. This study was reviewed and approved by the VASDHS Institutional Review Board (code: H130289). Patient consent was waived by the VASDHS Institutional Review Board due to the retrospective study design.

### 2.2. DIMM “Tune Up” Clinic Intervention

As previously described [11], the DIMM “tune up” clinic is a pharmacist-run CMM clinic for complex patients at the VASDHS with type 2 diabetes and comorbid conditions. It was implemented in 2009 as a collaboration between the endocrinology and pharmacy departments and was overseen by an endocrinologist. Selection and allocation of patients for the DIMM “tune up” clinic were completed through referral by PCPs within VASDHS as part of routine practice, with referrals placed typically when patients exceeded HbA1c of 9%, but ultimately at the discretion of the referring provider. The goal was to help patients achieve their personalized glycemic control goals within a few one-hour visits by providing individualized care that was co-created with the patient. The “tune up” model of care was a multipronged strategy to provide patients with specific tools to help them make life-long durable changes and to achieve their personalized glycemic control. When glycemic control was achieved, patients were discharged from the DIMM “tune up” clinic back to their PCP for their routine care. 

### 2.3. DIMM-Managed Sample

We included adult patients (18 years and older) with a diagnosis of type 2 diabetes who were referred to the DIMM “tune up” clinic and had achieved their HbA1c goals, which we labelled as the DIMM-managed group. Additionally, patients who were discharged from the DIMM “tune up” clinic had to have attended at least one follow-up appointment with their primary care provider within 6 months of discharge. Since our objective was to compare the DIMM-managed group to the PCP-managed group, patients were excluded if they were seen by an endocrinologist for their diabetes care.

### 2.4. Control Group and Matching Strategy

Patients in the PCP-managed group (control) were included if they were 18 years or older, had a diagnosis of type 2 diabetes and had a visit with a VA primary care provider on the same day as a DIMM group patient, during the same period as the study group. Similar to the study DIMM-managed group, we excluded patients who were seen by an endocrinologist for their diabetes care. 

Patients in the PCP-managed group were seen in the primary care clinics; however, we did not categorize the patients by the type of provider who was seen. At the VA, the primary care clinics are staffed by physicians, nurse practitioners, and physician assistants. Everett and colleagues reported no differences in diabetes intermediate outcomes between physicians, nurse practitioners, and physician assistants at VA medical centers [19]. We assumed that there were no differences in the quality of care delivered by these provider types.

Patients who were enrolled in the DIMM “tune up” clinic were matched 1:1 to patients in the PCP-managed group using propensity scores methods [20]. Propensity scores were generated using the nearest neighbor approach, without replacement, and a caliper of 0.01 precision. Patients were matched based on age, sex, baseline HbA1c, baseline eGFR, baseline FPG, liver disease, renal failure, and DIMM “tune up” clinic entry (baseline) date. The baseline date represented the first consultation with a CPS at the DIMM “tune up” clinic for the treatment group and matched to a corresponding primary care appointment date with a PCP-managed patient. 

**Figure 1:**
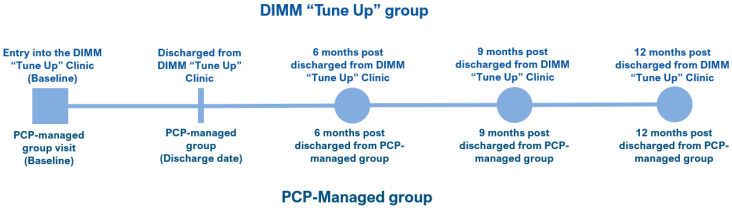
DIMM-managed group and PCP-managed group study timeline.

Per DIMM “tune up” clinic protocol, the goal was for patients to achieve their personalized HbA1c goal within 6 months of treatment; thus, the “treatment” period for the control group was set at 6 months of usual PCP care after the clinic entry (baseline) date. The index (discharge) date was defined as the date when the DIMM-managed group patient was discharged, at goal, from the clinic. This definition was applied to the corresponding matched PCP-managed patient. Starting from the discharge date, data were collected 6-, 9-, and 12-months post-discharge (Figure 1).

### 2.5. Variables

Baseline characteristics of the cohorts included the following: age, gender, BMI, baseline laboratory values (hemoglobin A1c (HbA1c), low density lipoprotein (LDL), high density lipoprotein (HDL), triglyceride (TG), fasting plasma glucose (FPG), and estimated glomerular filtration rate (eGFR)), and comorbidities (congestive heart failure, cardiac arrhythmias, valvular disease, pulmonary circulation disorder, peripheral vascular disease hypertension, paralysis, other neurologic disorder, chronic pulmonary disease, thyroid disorder, renal failure, liver disease, peptic ulcer disease, human immunodeficiency virus or acquired immunodeficiency syndrome, lymphoma, metastatic cancer, tumor without metastasis, rheumatoid arthritis, coagulopathy, obesity, depression, bipolar disorder, generalized anxiety disorder, schizophrenia, and posttraumatic stress disorder). 

Personalized therapeutic goals were assigned based on the most current national diabetes, hypertension, and lipid guidelines at the time of treatment, and further tailored to the patients’ age, comorbidities, duration of diabetes, hypoglycemia risk, and fall risk. Data points were retrieved from the VA patient electronic medical record system using a 3-month window, depending on availability of laboratory data, and missing values were addressed using the last observation carried forward approach. The main outcomes included HbA1c, LDL, HDL, and FPG at date of entry into the clinic, discharge date from the clinic, 6 months post-discharge, 9 months post-discharge, and 12 months post-discharge. For the secondary aims, dichotomous variables (Yes/No) for attainment of specific laboratory goals were generated. These included achieving HbA1c < 7%, HbAc1 < 8%, HbA1c < 9%, FPG between 70 to 130 mg/dL, LDL < 70 mg/dL, LDL < 100 mg/dL, and HDL > 40 mg/dL. 

### 2.6. Statistical Analysis

Baseline comparison between groups was performed using the independent *t* test for continuous data and the chi-square test for discrete data. Bivariate analyses were performed to compare the metabolic biomarkers at all time points. Comparison of metabolic biomarker (HbA1c, FPG, LDL, and HDL) levels at date of entry (baseline) into the clinic, discharge date, 6 months post-discharge, 9 months post-discharge, and 12 months post-discharge were performed using independent t tests at each period between the DIMM-managed and PCP-managed groups. 

For the primary aim, we constructed generalized estimating equations (GEE) with multivariable linear regression models to evaluate the impact of the DIMM “tune up” clinic on metabolic biomarkers (HbA1c, FPG, LDL, and HDL) compared to the PCP group, controlling for time, age, sex, BMI, and the number of comorbidities. Autoregressive correlation was used for the covariance structure, and clustered robust standard errors were estimated at the patient level. Marginal effects were estimated along with the corresponding 95% confidence interval (CI). For the secondary aims, chi-square tests were performed to evaluate the differences in the proportion of patients who achieved specific laboratory outcomes (HbA1c < 7%, HbAc1 < 8%, HbA1c < 9%, FPG between 70 to 130 mg/dL, LDL < 70 mg/dL, LDL < 100 mg/dL, and HDL > 40 mg/dL) at 6 months, 9 months, and 12 months post-discharge from the clinic.

We performed a sensitivity analysis using multiple imputation methods to handle missing data. We used 5 imputations based on the multivariate normal distribution, which uses Markov chain Monte Carlo simulations to fill in the missing data [21,22]. We assumed that the data were missing at random, and we visualized the missing patterns for violation of this assumption. We compared the results of the imputed results to the base-case GEE models. Statistical significance was defined a priori as a two-tailed alpha <0.05. All analyses were performed using Stata SE 17 (Stata Corp., College Station, TX, USA).

## 3. Results

### 3.1. Demographics

There were 123 propensity score-matched subjects in each of the DIMM-managed and PCP-managed groups. Of the 2432 PCP-managed candidates that met our inclusion and exclusion criteria, 123 patients were selected after 1:1 matching. Baseline comorbidity data within the past 5 years of each patient’s baseline visit were compiled and compared between the two groups (Table 1). Propensity score matching resulted in a balanced set of observed characteristics for the study cohorts. There were no statistically significant differences reported between the two groups. At baseline, the DIMM-managed group had an average age of 60.9 years, HbA1c of 10.2%, LDL of 86.8 mg/dL, and HDL of 40.0 mg/dL. Similarly, the PCP-managed group had an average age of 60.4 years, HbA1c of 10.3%, LDL of 94.4 mg/dL, and HDL of 42.6 mg/dL. 

### 3.2. Metabolic Biomarker Comparisons across Time

**Figure 2:**
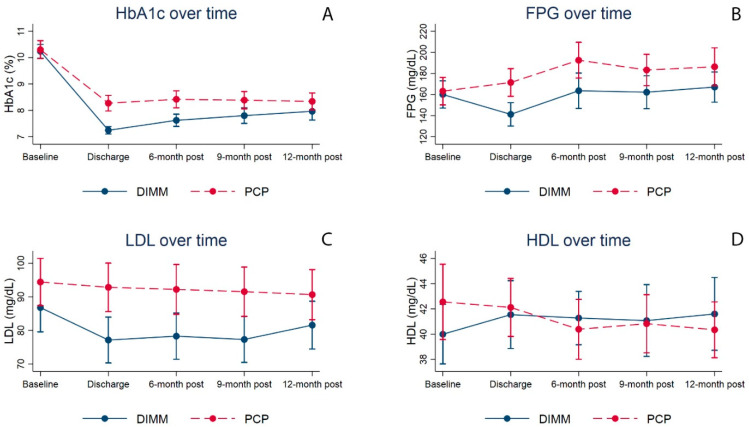
Comparisons of average metabolic biomarker levels between the DIMM-managed and PCP-managed groups across the study period, including 6-, 9-, and 12-months post-discharge. Shown are the trends of mean hemoglobin A1c (**A**), fasting plasma glucose (**B**), low-density lipoprotein (**C**), and high-density lipoprotein (**D**) for patients in the DIMM-managed and PCP-managed groups at entry in clinic, discharge from clinic, 6 months post-discharge, 9 months post-discharge, and 12 months post-discharge.

Figure 2 illustrates the trends for HbA1c, FPG, LDL, and HDL from baseline, discharge, and 6 months, 9 months, and 12 months post-discharge for both groups. DIMM-managed patients achieved an average HbA1c reduction of 3%-points upon discharge and maintained an HbA1c concentration that was significantly lower than PCP-managed patients at 6 months (7.62% versus 8.42%; *p* < 0.001) and 9 months (7.80% versus 8.39%; *p* = 0.009) post-discharge (Table 2). Although DIMM-managed patients had lower HbA1c than PCP-managed patients at 12 months post-discharge, the difference was not statistically significant (7.97% versus 8.34%; *p* = 0.105). 

DIMM-managed patients achieved an average FPG that was significantly lower than PCP-managed patients at discharge (141.25 mg/dL versus 171.43 mg/dL; *p* < 0.001) and at 6 months (163.63 mg/dL versus 192.58 mg/dL; *p* = 0.017) post-discharge. Although DIMM-managed patients had lower FPG at 9 months and 12 months post-discharge, the difference was not statistically significant.

DIMM-managed patients achieved an average LDL that was significantly lower than PCP-managed patients at discharge (77.13 mg/dL versus 92.83 mg/dL; *p* = 0.002), 6 months (78.27 mg/dL versus 92.21 mg/dL; *p* = 0.007), and 9 months (77.30 mg/dL versus 91.54 mg/dL; *p* = 0.005) post-discharge. Although DIMM-managed patients had lower LDL at 12 months post-discharge, the difference was not statistically significant. No significant differences in HDL levels were reported between DIMM-managed and PCP-managed patients throughout the follow-up period. 

### 3.3. Metabolic Goal Attainment across Time

A significantly larger proportion of DIMM-managed patients maintained HbA1c <8% compared to PCP-managed patients at 6 months (67.5% versus 47.2%, *p* = 0.001) and 9 months (62.6% versus 49.6%, *p* = 0.040) post-discharge; DIMM-managed patents had a larger but non-significant proportion at goal compared to PCP-managed patients at 12 months (56.9% versus 47.2%, *p* = 0.126) post-discharge (Table 3). Similarly, a significantly larger proportion of DIMM-managed patients sustained HbA1c < 9% compared to PCP-managed patients at 6 months (87.8% versus 66.7%, *p* < 0.001) and 9 months (82.1% versus 68.3%, *p* = 0.012) post-discharge; however, there was no significant difference at 12 months. Although DIMM-managed patients had a significantly higher proportion of patients with HbA1c < 7% compared to the PCP-managed patients (*p* = 0.005) at discharge, the difference was not significant at 6 months (*p* = 0.197), 9 months (*p* = 0.197), and 12 months (*p* = 0.658) post-discharge. Notably, due to the complex nature of the patient population, the individualized target A1C goals tended to be higher than 7%, especially in older more complex patients. A larger proportion of DIMM-managed patients maintained FPG between 70–130 mg/dL compared to PCP-managed patients at 12 months post-discharge (*p* = 0.022). Patients in the DIMM-managed clinic had a significantly higher proportion who achieved LDL < 70 mg/dL at discharge (50.0% versus 30.3%; *p* = 0.002) and 9 months (44.7% versus 32.3%); *p* = 0.045) post-discharge. Patients in the DIMM-managed group had a higher proportion who achieved LDL < 100 mg/dL through 12 months post-discharge compared to PCP-managed patients (77.2% versus 64.5%; *p* = 0.028). There was no significant difference in the proportion achieving HDL > 40 mg/dL at any post-discharge time point.

### 3.4. GEE Model Results

Findings from the base-case GEE models were similar to the bivariate analyses (Appendix A). DIMM-managed patients had a greater reduction in HbA1c at 6 months (−0.72%; 95% CI: −1.12%, −0.31%) and 9 months (−0.55%; 95% CI: −0.99%, −0.10%) post-discharge compared to the PCP-managed group, controlling for baseline confounders. DIMM-managed patients had a greater reduction in FPG at 6 months (−24.02 mg/dL; 95% CI: −48.03, −0.01) post-discharge compared to the PCP-managed group, controlling for baseline confounders. DIMM-managed patients had a greater reduction in LDL at 6 months (−15.42 mg/dL; 95% CI: −25.12, −5.71) and 9 months (−15.10 mg/dL; 95% CI: −24.87, −5.33) post-discharge compared to the PCP-managed group, controlling for baseline confounders. No significant differences in marginal effects were reported for HDL. 

### 3.5. Sensitivity Analysis

There were 6.9% missing data for the baseline BMI, 6.5% missing data for the baseline LDL, and 1.2% missing data for the LDL at 6 months post-discharge. Additional missing data were observed, but they made up <1% of the total sample. There did not appear to be any patterns to the missing data. In the sensitivity analysis where we used multiple imputations to handle missing data, we reported that that FPG levels at 9 months post-discharge were statistically different between the DIMM-managed and PCP-managed groups (Appendix B). This contradicted the base-case GEE model results, which reported that there was no significant difference at 9-months post-discharge. For all other comparisons, the results between the inputted data and base-case GEE models were similar. 

## 4. Discussion

While the literature contains studies and meta-analyses reviewing the beneficial impact of pharmacist-led CMM efforts on improving diabetes care, HbA1c reduction is typically ≤1%, and no long-term follow up is evaluated once patients are discharged [11,14,17,23,24,25]. One study, which included a sustainability evaluation following one year of CMM-type services, found that 12 months post-discharge, patients experienced a significant drop in the proportion who had achieved optimal HbA1c goals to nearly baseline values [26]. Our study adds to the paucity of literature exploring the outcomes or sustainability of glycemic control post-discharge from a pharmacist-run diabetes clinic. The findings from our study are reflective of veteran patients, in that they were older and had uncontrolled diabetes, with concurrent complex comorbidities such as obesity, hypertension, hyperlipidemia, heart failure, and COPD being the conditions of highest prevalence, along with mental health disorders, with depression being the most common (Table 1). Typically, patients with higher comorbidities and a higher number of medications are associated with non-adherence and poor glycemic control [27,28,29,30]. Moreover, mental health disorders may add an additional challenge in controlling diabetes, and depression has been identified as a significant correlate of medication non-adherence and suboptimal diabetes control [31]. 

Compared to the PCP-managed group, patients treated in the DIMM “tune up” model clinic achieved a significant HbA1c reduction of 3% upon discharge. Following discharge, the DIMM-managed group maintained significantly better long-term HbA1c control for up to 9 months, compared to patients in the PCP-managed group. Similar patterns were reported for other metabolic biomarkers (FPG and LDL). We observed that the difference in metabolic biomarkers between the two groups decreased over time. Although statistical differences were not realized at 12 months post-discharge, the DIMM-managed group had a lower HbA1c of 7.97% compared to 8.34% for the PCP-managed group, which suggests a clinically meaningful difference. 

A larger proportion of patients in the DIMM-managed group also maintained an HbA1c of <8%, compared to patients in the PCP-managed group, at 6, 9, and 12 months post-discharge. Although the absolute difference in the proportion who achieved HbA1c < 8% was 10% at 12 months post-discharge, favoring the DIMM-managed group compared to the control, the difference did not reach statistical significance (57% versus 47%, *p* > 0.05). Achieving this amount of glycemic control suggests a clinically meaningful finding for patients, as lower HbA1c is associated with the prevention of long-term complications [8], as well as for health-systems, which may be evaluated based on achieving metabolic performance measures or benchmarks. The US Veterans Health Administration uses a performance measure of <9%, due to the complex nature of veteran patients [32]. For this category, a significantly larger proportion of DIMM-managed patients sustained HbA1c < 9%, compared to PCP-managed patients, at 6 months (87.8% versus 66.7%, *p* < 0.001) and 9 months (82.1% versus 68.3%, *p* = 0.012) post-discharge; however, there was no significant difference at 12 months post-discharge. Similarly, DIMM-managed patients had a higher proportion of patients who achieved a metabolic goal of LDL < 100 mg/dL compared to PCP-managed patients at 6-, 9- and 12-months post-discharge. 

The DIMM “tune up” clinic uses the CMM model with a special emphasis on coupling personalized clinical care with patient empowerment through diabetes education, therapeutic lifestyle, and adherence optimization tools to achieve and maintain glycemic control beyond the treatment course. Spending more time (an average of three 60-min visits over the initial 6 months), using motivational interviewing, as well as creating a positive space for nonjudgmental discussions were additional key elements to empower patients to take an active role in their care. Our study suggests that these patient-specific behavioral tools provided in the “tune up” model may contribute to effective long-term therapeutic lifestyle changes and patient’s self-efficacy in the chronic management of type 2 diabetes. These positive long-term results are consistent with the short-term success shown in previous studies of this DIMM clinic [10,11,12,14]. More importantly, our findings address the gap in the evaluation of the long-term effectiveness of the DIMM “tune up” clinic up to 9 months after patients have been released back to their primary care provider, showing that the progress was sustained in most of the glycemic and metabolic end points. Results and methods from this study can inform future evaluation of long-term durability of results of collaborative pharmacist-led CMM clinics focused on treating patients with diabetes. 

Overall, patients treated in the DIMM “tune up” model clinic successfully achieved and maintained glycemic control through 9 months post-discharge compared to the PCP-managed patients. However, based on the 12-month post-discharge data, we postulate that this attenuation in metabolic biomarkers among DIMM-managed patients may necessitate or require an additional touch point by either phone, video, or an in-person visits with the pharmacist following discharge in order to maintain long-term glycemic control. Nine months post-discharge may offer the best timing for follow up. This conjecture is supported by a previous study, where only patients who continued to see the pharmacist at 9 months in a clinic, using what would now be called a CMM model for hypertension, had significantly better blood pressure control compared to those who had returned to usual PCP care prior to 9 months [33]. With the general acceptance of telemedicine due to the pressures imposed by the COVID-19 pandemic, access to the DIMM “tune up” clinic could be improved by making a 9-month post-discharge follow-up visit convenient and straightforward [34,35,36].

### Limitations

There are several limitations of this study that should be considered. First, this was a retrospective cohort study with a matched control group. We used propensity score matching methods to balance the observable covariates; however, there is still a potential for imbalance among the unobserved covariates that could generate biased estimates of the treatment effect. Additionally, the clinic in its design was limited to a half-day per week, thereby reducing our ability to obtain a larger experimental cohort. Despite this limitation, we were able to identify several metabolic markers that were significant at post-discharge from the DIMM “tune up” clinic. Moreover, there were missing data among the metabolic biomarkers. We performed a sensitivity analysis using multiple imputation methods; findings were similar to the base-case GEE model, but FPG at 9 months post-discharge was significantly different using the imputed dataset. This could be due to random error, but future investigation should make a greater effort to acquire a complete dataset. Next, the DIMM clinic incorporated multiple elements to empower patients to take control of their disease and maintain long-term metabolic control. However, we did not investigate which of these element had the greatest effect on outcomes. Lastly, this study was performed on the veteran population, which is different than the general population. Veteran patients tend to have more complex comorbidities and to be older, with increased mental health diagnoses [37,38]. Large, integrated healthcare systems, similar to the VA, may find the results of our study informative as they plan to implement their own pharmacist-led DIMM “tune up” clinic model. 

## 5. Conclusions

This study provides additional empirical evidence that the DIMM “tune up” clinic model, using a unique CMM approach addressing personalized lifestyle, medication adherence, empowerment, and clinical care, was effective at achieving and sustaining glycemic goals for HbA1c and LDL levels up to 9 months after being discharged from the DIMM “tune up” clinic. DIMM-managed patients maintained a higher proportion of HbA1c (<8% and <9%) and FPG goals up to 9 months post-discharge, compared to patients in a comparison PCP-managed group. The outcomes suggest that sustaining these effects beyond 9 months post-discharge may require an additional follow-up visit or touch point. Additional studies evaluating long-term outcomes following CMM care are needed.

## Figures and Tables

**Table 1:** Demographics and clinical characteristics of DIMM clinic and PCP comparison group at baseline (matched by age, HbA1c baseline, and entry date).

Characteristics	DIMM-Managed Group n = 123	PCP-Managed Group n = 123	*p*-Value ^a^	Missing Data (DIMM, PCP)
Age (years)	60.9 (8.3)	60.4 (11.7)	0.740	0, 0
BMI (kg/m^2^)	33.1 (6.2)	32.7 (6.7)	0.641	10, 7
Male, n (%)	119 (96.8%)	117 (95.1%)	0.518	0, 0
HbA1c (%), mean (SD)	10.2 (1.4)	10.3 (1.9)	0.779	0, 0
LDL (mg/dL), mean (SD)	86.8 (39.4)	94.4 (37.8)	0.136	9, 7
HDL (mg/dL), mean (SD)	40.0 (13.2)	42.6 (16.6)	0.184	2, 0
TG (mg/dL), mean (SD)	235.5 (204.1)	244.1 (364.0)	0.822	4, 0
FPG (mg/dL), mean (SD)	160.1 (72.3)	163.3 (72.8)	0.732	0, 0
eGFR (mL/min), mean (SD)	80.0 (28.7)	81.3 (30.7)	0.735	0, 0
Congestive heart failure, n (%)	19 (15.5%)	14 (11.4%)	0.350	0, 0
Cardiac arrhythmias, n (%)	23 (18.7%)	19 (15.5%)	0.498	0, 0
Valvular disease, n (%)	7 (5.7%)	4 (3.3%)	0.355	0, 0
Pulmonary circulation disorder, n (%)	8 (6.5%)	3 (2.4%)	0.123	0, 0
Peripheral vascular disease, n (%)	20 (16.3%)	15 (12.2%)	0.361	0, 0
Hypertension (uncomplicated), n (%)	113 (91.9%)	108 (87.8%)	0.291	0, 0
Hypertension (complicated), n (%)	14 (11.4%)	13 (10.6%)	0.838	0, 0
Paralysis, n (%)	3 (2.4%)	3 (2.4%)	>0.999	0, 0
Other neurologic disorder, n (%)	4 (3.3%)	5 (4.1%)	0.734	0, 0
Chronic pulmonary disease, n (%)	29 (23.6%)	20 (16.3%)	0.151	0, 0
Thyroid, n (%)	12 (9.8%)	10 (8.1%)	0.655	0, 0
Renal failure, n (%)	19 (15.5%)	14 (11.4%)	0.350	0, 0
Liver disease, n (%)	23 (18.7%)	24 (19.5%)	0.871	0, 0
Peptic ulcer disease, n (%)	2 (1.6%)	1 (0.8%)	0.561	0, 0
AIDS/HIV, n (%)	1 (0.8%)	1 (0.8%)	>0.999	0, 0
Lymphoma, n (%)	0 (0.0%)	0 (0.0%)	N/A	0, 0
Metastatic cancer, n (%)	2 (1.6%)	1 (0.8%)	0.561	0, 0
Tumor without metastasis, n (%)	10 (8.1%)	15 (12.2%)	0.291	0, 0
Rheumatoid arthritis, n (%)	3 (2.4%)	0 (0.0%)	0.081	0, 0
Coagulopathy, n (%)	4 (3.3%)	3 (2.4%)	0.701	0, 0
Obesity, n (%)	75 (61.0%)	68 (55.3%)	0.366	0, 0
Depression, n (%)	52 (42.3%)	55 (44.7%)	0.700	0, 0
Bipolar, n (%)	9 (7.3%)	9 (7.3%)	>0.999	0, 0
Generalized anxiety disorder, n (%)	6 (4.9%)	7 (5.7%)	0.776	0, 0
Schizophrenia, n (%)	1 (0.8%)	3 (2.4%)	0.313	0, 0
PTSD, n (%)	3 (2.4%)	7 (5.7%)	0.197	0, 0

Abbreviations: HbA1c, glycosylated hemoglobin; BMI, body mass index; DIMM, diabetes intense medical management; FBP, fasting plasma glucose; HDL, high-density lipoprotein; LDL, low-density lipoprotein; PCP, primary care provider; TG, triglycerides; PTSD, posttraumatic stress disorder. ^a^ *t* test for means; chi-square test for proportions.

**Table 2:** Glycemic and metabolic measures for the DIMM “tune up” clinic and PCP-managed groups at baseline, discharge (index date), 6-, 9-, and 12-months post-discharge.

End points	DIMM-Managed Group (n = 123)	PCP-Managed Group (n = 123)	*p*-Value ^a^	Missing Data (DIMM, PCP)
HbA1c (%), mean (SD)				
Baseline	10.24 (1.45)	10.30 (1.92)	0.780	0, 0
Discharge	7.25 (0.79)	8.27 (1.67)	<0.001	0, 0
6 months	7.62 (1.30)	8.42 (1.82)	<0.001	0, 0
9 months	7.80 (1.65)	8.39 (1.84)	0.009	0, 0
12 months	7.97 (1.85)	8.34 (1.78)	0.105	0, 0
FPG (mg/dL), mean (SD)				
Baseline	160.09 (72.31)	163.26 (72.79)	0.732	0, 0
Discharge	141.25 (61.91)	171.43 (73.17)	<0.001	0, 0
6 months	163.63 (93.97)	192.58 (94.78)	0.017	0, 0
9 months	162.26 (87.80)	183.30 (83.21)	0.055	0, 0
12 months	167.10 (79.89)	186.34 (100.42)	0.098	0, 0
LDL (mg/dL), mean (SD)				
Baseline	86.80 (39.40)	94.42 (37.80)	0.136	9, 7
Discharge	77.13 (38.00)	92.83 (39.73)	0.002	4, 1
6 months	78.27 (38.44)	92.21 (41.09)	0.007	2, 1
9 months	77.30 (38.18)	91.54 (40.61)	0.005	2, 0
12 months	81.56 (39.94)	90.66 (41.41)	0.082	2, 0
HDL (mg/dL), mean (SD)				
Baseline	39.99 (13.19)	42.55 (16.59)	0.184	2, 0
Discharge	41.54 (15.07)	42.12 (12.77)	0.750	2, 0
6 months	41.28 (11.81)	40.39 (13.26)	0.579	1, 0
9 months	41.07 (15.96)	40.83 (12.86)	0.895	1, 0
12 months	41.60 (16.16)	40.34 (12.34)	0.494	1, 0

Abbreviations: HbA1c, glycosylated hemoglobin; FPG, fasting plasma glucose; LDL, low-density lipoprotein; HDL, high-density lipoprotein; post-DC, post-discharge; ^a^ independent *t* test.

**Table 3:** Therapeutic goal achievement at 6, 9, and 12 months post-discharge.

End Points	Baseline			Discharge			6-Months Post-Discharge			9-Months Post-Discharge			12-Months Post-Discharge		
Metabolic Goals	DIMM	PCP	*p*-Value ^a^	DIMM	PCP	*p*-Value ^a^	DIMM	PCP	*p*-Value ^a^	DIMM	PCP	*p*-Value ^a^	DIMM	PCP	*p*-Value ^a^
HbA1c < 7, n (%)	0 (0.0%)	0 (0.0%)	n/a	45 (36.6%)	25 (20.3%)	0.005	38 (30.9%)	29 (23.6%)	0.197	38 (30.9%)	29 (23.6%)	0.197	32 (26.0%)	29 (23.6%)	0.658
HbA1c < 8, n (%)	0 (0.0%)	0 (0.0%)	n/a	101 (82.1%)	61 (49.6%)	<0.001	83 (67.5%)	58 (47.2%)	0.001	77 (62.6%)	61 (49.6%)	0.040	70 (56.9%)	58 (47.2%)	0.126
HbA1c < 9, n (%)	18 (14.6%)	35 (28.5%)	0.008	123 (100.0%)	85 (69.1%)	<0.001	108 (87.8%)	82 (66.7%)	<0.001	101 (82.1%)	84 (68.3%)	0.012	97 (78.9%)	85 (69.1%)	0.081
FPG = 70–130, n (%)	41 (33.3%)	39 (31.7%)	0.785	58 (47.2%)	36 (29.3%)	0.004	47 (38.2%)	33 (26.8%)	0.057	50 (40.7%)	33 (26.8%)	0.022	50 (40.7%)	33 (26.8%)	0.022
LDL < 70, n (%)	42 (36.2%)	29 (23.4%)	0.077	61 (50.0%)	36 (30.3%)	0.002	49 (40.2%)	41 (33.9%)	0.311	55 (44.7%)	39 (32.3%)	0.045	50 (40.7%)	40 (33.1%)	0.219
LDL < 100, n (%)	80 (69.0%)	68 (59.7%)	0.140	104 (85.3%)	75 (63.0%)	<0.001	100 (82.0%)	75 (62.0%)	0.001	103 (83.7%)	77 (63.6%)	<0.001	95 (77.2%)	78 (64.5%)	0.028
HDL > 40, n (%)	52 (42.3%)	61 (50.4%)	0.203	61 (49.6%)	65 (53.7%)	0.519	60 (48.8%)	55 (45.1%)	0.562	56 (45.5)	58 (47.5%)	0.752	62 (50.4%)	57 (46.7%)	0.564

Abbreviations: HbA1c, glycosylated hemoglobin; FPG, fasting plasma glucose; LDL, low-density lipoprotein; HDL, high-density lipoprotein; ^a^ chi-square test.

## Data Availability

The data presented in this study are available on request from the corresponding author. The data are not publicly available due to the confidentiality of the veterans in our care.

## Appendix A. GEE Model Results (Primary Aim Analysis)

**Table A1:** Results from the generalized estimating equation models.

Outcome	Time	Marginal Effect (95% CI)	*p*-Value
HbA1c	Entry	−0.04 (−0.47, 0.39)	0.861
HbA1c	Discharge	−0.96 (−1.30, −0.63)	<0.001
HbA1c	6-month post	−0.72 (−1.12, −0.31)	<0.001
HbA1c	9-month post	−0.55 (−0.99, −0.10)	0.016
HbA1c	12-month post	−0.32 (−0.79, 0.14)	0.171
FPG	Entry	−3.93 (−22.91, 15.06)	0.685
FPG	Discharge	−28.38 (−46.00, −10.76)	0.002
FPG	6-month post	−24.02 (−48.03, −0.01)	0.050
FPG	9-month post	−18.33 (−40.52, 3.85)	0.105
FPG	12-month post	−16.20 (−39.87, 7.47)	0.180
LDL	Entry	−10.71 (−20.83, −0.59)	0.038
LDL	Discharge	−17.59 (−27.70, −7.49)	0.001
LDL	6-month post	−15.42 (−25.12, −5.71)	0.002
LDL	9-month post	−15.10 (−24.87, −5.33)	0.002
LDL	12-month post	−9.82 (−19.97, 0.33)	0.058
HDL	Entry	0.12 (3.16, 3.40)	0.942
HDL	Discharge	0.98 (−2.61, 4.58)	0.592
HDL	6-month post	1.55 (−1.62, 4.71)	0.339
HDL	9-month post	1.23 (−2.56, 5.03)	0.524
HDL	12-month post	2.12 (−1.69, 5.93)	0.276

## Appendix B. GEE Models Using the Imputed Dataset

**Table A2:** Results from the generalized estimating equation models using multiple imputations.

Outcome	Time	Marginal Effect (95% CI)	*p*-Value
HbA1c	Entry	−0.05 (−0.47, 0.37)	0.813
HbA1c	Discharge	−1.02 (−1.35, 0.69)	<0.001
HbA1c	6-month post	−0.79 (−1.18, −0.39)	<0.001
HbA1c	9-month post	−0.57 (−1.01, −0.14)	0.009
HbA1c	12-month post	−0.37 (−0.81, 0.08)	0.109
FPG	Entry	−3.80 (−21.86, 14.27)	0.68
FPG	Discharge	−30.80 (−47.83, −13.78)	<0.001
FPG	6-month post	−29.57 (−52.90, −6.24)	0.013
FPG	9-month post	−21.67 (−43.01, −0.32)	0.047
FPG	12-month post	−19.87 (−42.48, 2.74)	0.085
LDL	Entry	−9.99 (−19.91, −0.07)	0.048
LDL	Discharge	−16.47 (−26.34, −6.60)	0.001
LDL	6-month post	−13.73 (−23.22, −4.24)	0.005
LDL	9-month post	−14.16 (−23.70, −4.62)	0.004
LDL	12-month post	−8.81 (−18.63, 1.00)	0.078
HDL	Entry	−2.19 (−5.99, 1.61)	0.259
HDL	Discharge	−0.37 (−3.86, 3.13)	0.837
HDL	6-month post	1.20 (−1.91, 4.32)	0.448
HDL	9-month post	0.44 (−3.20, 4.07)	0.813
HDL	12-month post	1.50 (−2.11, 5.11)	0.415
